# Presence of RD149 Deletions in *M. tuberculosis* Central Asian Strain1 Isolates Affect Growth and TNFα Induction in THP-1 Monocytes

**DOI:** 10.1371/journal.pone.0024178

**Published:** 2011-08-31

**Authors:** Akbar Kanji, Zahra Hasan, Mehnaz Tanveer, Raunaq Mahboob, Sana Jafri, Rumina Hasan

**Affiliations:** Department of Pathology and Microbiology, Aga Khan University, Karachi, Pakistan; Institut Pasteur, France

## Abstract

Central Asian Strain 1 (CAS1) is the prevalent *Mycobacterium tuberculosis* genogroup in South Asia. CAS1 strains carry deletions in RD149 and RD152 regions. Significance of these deletions is as yet unknown. We compared CAS1 strains with RD149 and concurrent RD149-RD152 deletions with CAS1 strains without deletions and with the laboratory reference strain, *M. tuberculosis* H37Rv for growth and for induction of TNFα, IL6, CCL2 and IL10 in THP-1 cells. Growth of CAS1 strains with deletions was slower in broth (RD149; p = 0.024 and RD149-RD152; p = 0.025) than that of strains without deletions. CAS1 strains with RD149 deletion strains further showed reduced intracellular growth (p = 0.013) in THP-1 cells as compared with strains without deletions, and also as compared with H37Rv (p = 0.007) and with CAS1 RD149-RD152 deletion strains (p = 0.029). All CAS1 strains induced higher levels of TNFα and IL10 secretion in THP-1 cells than H37Rv. Additionally, CAS1 strains with RD149 deletions induced more TNFα secretion than those without deletions (p = 0.013). CAS1 RD149 deletion strains from extrapulmonary sources showed more rapid growth and induced lower levels of TNFα and IL6 secretion in THP-1 cells than isolates from pulmonary sources. This data suggests that presence of RD149 reduces growth and increases the induction of TNFα in host cells by CAS1 strains. Differences observed for extrapulmonary strains may indicate an adaptation which increases potential for dissemination and tropism outside the lung. Overall, we hypothesise that RD149 deletions generate genetic diversity within strains and impact interactions of CAS1 strains with host cells with important clinical consequences.

## Introduction

Molecular epidemiological studies have suggested an association between *Mycobacterium tuberculosis* strains and geographical locations [1]. Predominant *M. tuberculosis* clades from the Indian subcontinent include Central Asian strains (CAS) defined by absence of spacers 4–7 and 23–34 [2], [3]. Within the CAS strains, CAS1_DEHLI (ST26) has been identified as being the most widespread (39%) in Pakistan [4]. Reasons underlying successful transmission of CAS genogroup strains in South Asia are unknown. Large sequence polymorphisms (LSPs) or regions of difference (RD) are identified as deletions or large sequence polymorphisms in *M. tuberculosis* strains.

Amongst *M. tuberculosis* isolates from Pakistan, deletions in RD149 are reported in 39.8% and concurrent RD149 and RD152 in 18.8% of CAS1 strains [5]. The impact of RD149 and RD152 deletions on *M. tuberculosis* biology is as yet unclear. RD149 region is known to consist of probable phage proteins (*Rv1572c-Rv1585c*), although the role of phage proteins has not been defined. RD152 region also consists of putative transposases (*Rv1756c, Rv1764*) along with virulence factor, *plcD* (*Rv1755c*) [6], [7]. Both RD149 and RD152 deletions are also known to be characteristic of Beijing genogroup strains which are prevalent worldwide, thought to be highly virulent [7] and also associated with multi-drug resistance [4], [8]. Clinical presentations of tuberculosis have been shown to differ depending on the infective *M. tuberculosis* strain group; Euro-American strains are associated with increased lung consolidation, while the meningitis caused by the East Asian/Beijing lineage was associated with younger adults, more rapid disease progression and fewer leucocytes in the cerebrospinal fluid (CSF) [9].

*In vitro* studies have shown that virulent *M. tuberculosis* Beijing clinical isolates grow more rapidly than H37Rv in murine and human macrophages as well as in THP-1 cell line model [8], [10], [11].

*M. tuberculosis* infection of macrophages has been shown to induce both proinflammatory cytokines; Tumor necrosis factor alpha (TNFα), Interleukin 2 (IL2) and Interleukin 6 (IL6) as well as downregulatory cytokine; Interleukin 10 (IL10) [12]. TNFα is essential for macrophage activation and granuloma formation [13], [14]. The release of TNFα in human macrophages infected with *M. tuberculosis* has the dual effect of increasing anti-mycobacterial activity by activating macrophages and inducing granuloma formation, while excess TNFα results in host cell necrosis and dissemination of mycobacteria [15]. TNFαalso increases the expression of chemokine CCL2 (monocyte chemoattractant protein (MCP-1)) by macrophages [16]. CCL2 plays a role in granuloma formation [17] and is reported to contribute towards protection against tuberculosis in the murine model [18]. IL10 is important for regulating immunity in mycobacterial infections and while IL10 is associated with progressive disease [19], absence of IL10 results in lack of antimycobacterial immunity [20].

Previous studies have shown that virulent clinical *M. tuberculosis* strains grow faster and cause more necrosis than laboratory strain H37Rv in murine bone marrow-derived macrophages [10]. Beijing genotype strains induce lower levels of proinflammatory cytokines (TNFα, IL6, IL12) as compared with *M. tuberculosis* H37Rv in human monocyte derived macrophages [11], [21]. Overall, reduced pro-inflammatory responses have been associated with increased intracellular persistence of *M. tuberculosis* strains [11], [22], [23].

It is known that RD1 encodes for highly antigenic proteins thought to play an important role in immunomodulation of the host by *M. tuberculosis* [24]. These and smaller deletions in *M. tuberculosis* may contribute to adaptation of strains for improved interactions with the host. These include a polymorphism in the *pks 15/1* gene which encodes for a phenol glycolipid whereby the mutation in the glycolipid results in immunosuppression of host responses. A previous study has shown that deletion of Rv1519 in CH, an outbreak strain belonging to the CAS2 lineage resulted in a higher secretion of immunosuppressive IL10 and IL6, contributing to its persistence in the human population [25].

This study explored the biological relevance of RD149 and of concurrent RD149 and RD152 deletions on CAS1 strains. *In vitro* broth growth of the CAS1 strains with RD149 and concurrent RD149–152 deletions was compared with that of CAS1 strains without deletions and with H37Rv. Intracellular growth of these strains was investigated using the human acute monocytic leukemia cell line (THP-1) [26], [27], [28]. *Mycobacterium*-induced TNFα, IL6, CCL2 and IL10 secretion was also determined.

## Materials and Methods

### Ethics Statement

Approval for this study was taken from the Ethics Review Committee of the Aga Khan University, Pakistan.

### Mycobacterial strain selection

*M. tuberculosis* isolates used in this study were obtained from diagnostic specimens submitted to the clinical laboratory at the Aga Khan University and stored in the strain bank. All strains were subcultured once on Middlebrook 7H10 agar before being stored in aliquots in glycerol peptone broth (GPB) on beads at −70°C. Central Asian Strain1 (ST26) strains (n = 133) from the strain bank at the Aga Khan University previously screened for RD deletions were included [5]. Of these 53 had RD149 deletions alone and 25 strains showed concurrent RD149 and RD152 deletions [5]. From this latter pool, strains with RD149 (n = 9) and with concurrent RD149 and RD152 (n = 9) were randomly selected for this study along with 3 CAS1 strains without deletions in RD149 or RD152 regions. Each strain culture was established from a fresh aliquot of each stored strain. *M. tuberculosis* H37Rv reference strain obtained from American Type Culture Collection (ATCC) was used as a control.

### Growth in broth

Growth in broth was assessed using radiometric methods; Study strains were grown in 7H9 broth medium supplemented by 5% ADC (Difco Laboratories, Detroit, MI, USA). The strains were then inoculated onto Middlebrook 7H10 agar (Becton Dickinson, Sparks, MD, USA) incubated for a 3 further weeks. Bacterial colonies were suspended in 1 ml sterile PBS, vortexed vigorously for 30 sec to obtain a single cell suspension and matched with 0.5 McFarland (approx cell density is 1.5×10^8^/ml).

The bacterial suspension was diluted (1∶100) to a final concentration of 1.5×10^6^/ml, 0.1 ml of the final concentration was added to BACTEC 12B medium culture vials (Becton Dickinson, USA) containing ^14^C-labelled fatty acid substrate and incubated at 37°C for up to 37 days [29], [30]. The growth in broth for days 0 to 37 is mentioned in Figure S1. As peak growth was observed at Day 2 for all strains measurements at this interval were used for further analysis.

### Intracellular growth

*M. tuberculosis* were first grown in Middlebrook 7H9 broth medium supplemented by 5% ADC (Difco Laboratories, Detroit, MI, USA) at 37°C to mid-logarithmic phase (OD_620 nm_ = 0·5). Aliquots of mycobacteria at a stock concentration of 2×10^6^ CFU/ml in 7H9 broth medium containing 15% glycerol were stored at −70°C for use in monocyte infection assays as described previously [22].

The THP-1 monocyte cell line was obtained from the ATCC, USA. THP-1 cells grown in RPMI 1640 (Gibco BRL) supplemented with 10% fetal calf serum, 10 mmol/L HEPES and 2 mmol/L glutamine at 37°C in 5% CO_2_ [31]. THP-1 cells were plated at (2×10^5^ cells/ well) in 48 well tissue culture plates. The cells were differentiated by addition of 20 ng/ml Phorbol Ester Myristate (PMA, Sigma) and 5 ng/ml recombinant human interferon gamma (rhIFNγ, Endogen) for 24 hrs.

*M. tuberculosis* inoculum for infection was prepared as described previously [31]. Briefly, THP-1 cells (2×10^5^/well) were infected with *M. tuberculosis* (2×10^5^ CFU/ml) for 3 h (T^0^), the supernatants were collected and monolayers washed thoroughly with phosphate buffer saline (PBS) to remove extracellular adherent mycobacteria. For the T^0^ wells, THP-1 cells were lysed with 100 µl of 0.5% Triton X-100 and lysate plated on 7H10 agar plates for enumeration by colony forming unit (CFUs) counts.

Fresh RPMI medium was added to the remaining wells until harvested. Cell supernatants were collected at days 1 and 3 for cytokine measurements, were filtered using 0.2 µm filters, aliquoted and stored at −80°C until used. Cell lysates were plated for CFUs at 1, 3, 5 and 7 days post-infection. The intracellular growth pattern observed for each strain is depicted in Figure S2. The largest change in growth was observed between days 0 and 3. Therefore, analysis of intracellular growth for each strain was calculated using the ratio of growth at day3/day 0 for each isolate.

### Measurement of Cytokines and Chemokines

Cytokines (TNFα, IL6 and IL10) standards and monoclonal antibody pairs for capture and detection obtained from Endogen (Rockford, IL, USA) were used to obtain a dose response curve with a range of detection from 3.9–1000 pg/ml using a sandwich ELISA technique as described previously. Each well was measured in duplicate. Chemokine, (CCL2) standards and monoclonal antibody pairs for capture and detection obtained from R&D Systems (Abingdon, UK) were used to obtain a dose response curve with a range of detection from 6.25–1000 pg/ml for CCL2. All measurements were carried out according to the manufacturer's instructions and as reported previously [31], [32].

### Statistical analysis

Non–parametric statistical analysis was performed using the Statistical Package for Social Sciences software (SPSS version 17.0). The analyzed variables for growth indices and CFU/ml were compared using Mann-Whitney U Tests. While the analyzed variables for cytokine analysis was compared using one way ANOVA (Tukey, Post Hoc analysis) and Kruskal Wallis Test.

## Results

*M. tuberculosis* strains were isolated from both male (n = 9) as well as female (n = 12) patients, Table 1. The median ages of male (24.5 y) and female (24 y) patients was comparable, p = 0.36 as analysed using the Mann-Whitney U Test.

**Table 1:** Characteristics of *Mycobacterium tuberculosis* CAS1 genotype strains used in the study.

No	ID	Age	Gender	Strain	Source	RD deletion
1	Reference			H37Rv	ATCC	none
2	CAS1a	55	Male	CAS1 (ST26)	Sputum	none
3	CAS1b	16	Female	CAS1 (ST26)	Sputum	none
4	CAS1c	36	Female	CAS1 (ST26)	Sputum	none
5	S1	23	Female	CAS1 (ST26)	Sputum	RD149
6	S2	18	Female	CAS1 (ST26)	Sputum	RD149
7	S5	70	Male	CAS1 (ST26)	Sputum	RD149
8	S6	20	Female	CAS1 (ST26)	Sputum	RD149
9	EP1	27	Male	CAS1 (ST26)	CSF	RD149
10	EP2	5	Male	CAS1 (ST26)	CSF	RD149
11	EP4	39	Female	CAS1 (ST26)	CSF	RD149
12	EP7	29	Male	CAS1 (ST26)	Urine	RD149
13	EP10	25	Female	CAS1 (ST26)	CSF	RD149
14	S3	35	Female	CAS1 (ST26)	Sputum	RD149-RD152
15	S4	20	Male	CAS1 (ST26)	Sputum	RD149-RD152
16	S7	47	Female	CAS1 (ST26)	Sputum	RD149-RD152
17	S8	20	Male	CAS1 (ST26)	Sputum	RD149-RD152
18	S9	18	Female	CAS1 (ST26)	Sputum	RD149-RD152
19	S10	22	Male	CAS1 (ST26)	Sputum	RD149-RD152
20	EP6	17	Female	CAS1 (ST26)	CSF	RD149-RD152
21	EP8	52	Female	CAS1 (ST26)	CSF	RD149-RD152
22	EP9	60	Male	CAS1 (ST26)	CSF	RD149-RD152

Central Asian Strain 1, CAS1 (ST26) as identified by SpolDB4.0.

Source of samples: Sputum, Cerebrospinal Fluid (CSF), Urine and American Type Culture Collection.

‘None’ denotes strains which had no deletions in RD149 and RD152 regions.

### CAS1 clinical strains show variable growth in broth

**Figure 1:**
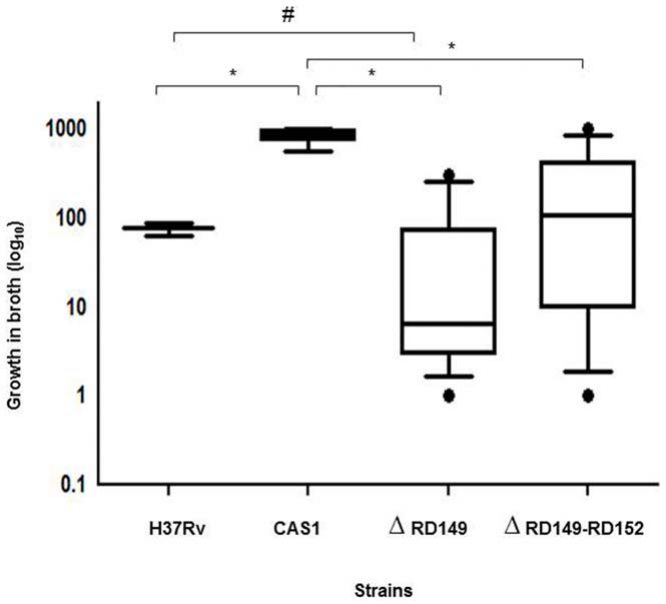
CAS1 strains with RD149 and concurrent RD149-RD152 deletions show slower growth in broth as compared with CAS1 (without deletions). The graph depicts the growth in broth of *M. tuberculosis* strains H37Rv, CAS1 (without deletions, n = 3), CAS1 strains with RD149 (n = 9) and concurrent RD149-RD152 (n = 9) deletions. Growth in broth was calculated as a ratio of growth at day 2/day 0. The box and whiskers plot represent the data between 10^th^ and 90^th^ quartiles, with the horizontal line indicating the median value. ‘*’ denotes significantly reduced growth (p<0.05) as compared with CAS1 (without deletions). ‘#’ denotes significantly reduced growth (p<0.05) as compared with H37Rv using the Mann-Whitney U test. *Δ* denotes deletions.

**Figure 2:**
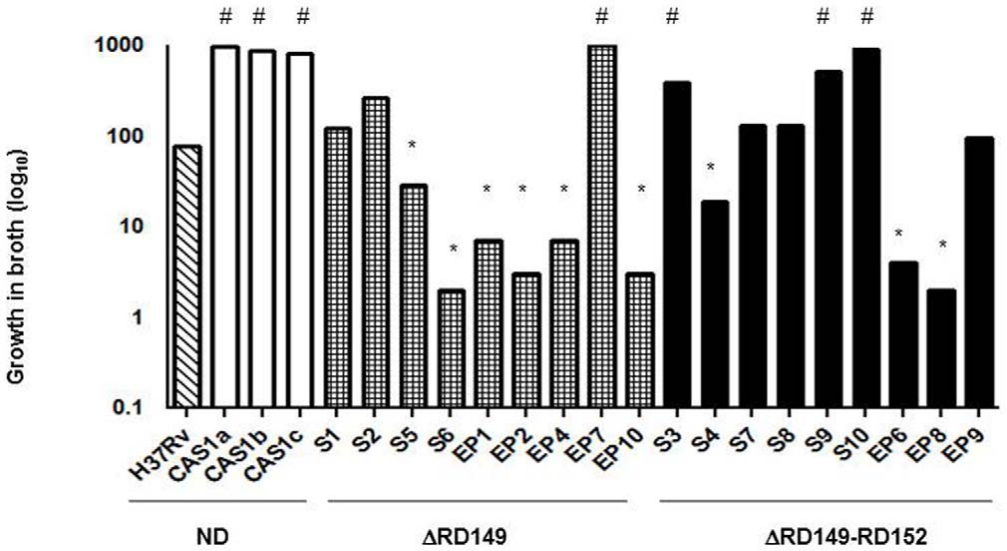
Differential growth of CAS1 strains with RD149 and concurrent RD149-RD152 deletions. The graph depicts the growth in broth of CAS1 strains without deletions (CAS1, n = 3), CAS1 with RD149 deletions (n = 9) and CAS1 strains with concurrent RD149-RD152 deletions (n = 9) as compared with the laboratory strain *M. tuberculosis* H37Rv. The bar graph represents the median and 95% confidence interval (CI) of each strain. ‘*’ denotes significantly reduced growth (p<0.05) as compared with H37Rv. ‘#’ denotes significantly increased growth (p<0.05) as compared with H37Rv using the Mann-Whitney U test. *Δ* denotes deletions; ND denotes no deletions.

Growth of CAS1 strains with deletions was slower in broth (RD149, p = 0.024; and RD149-RD152, p = 0.025) than that of strains without deletions. Comparison of CAS1 strains with *M. tuberculosis* H37Rv showed that while CAS1 strains without deletion strains grew more rapidly in broth as compared with *M. tuberculosis* H37Rv (p = 0.036), growth of H37Rv was faster than that of CAS1 strains with RD149 deletions (p = 0.04), Figure 1. When assessed individually however, the growth of deletion strains showed variability; One strain with RD149; and 3 strains with concurrent RD149-RD152 deletions grew faster, whereas 6 strains with RD149 and 3 strains with RD149-RD152 grew slower than the laboratory reference strain H37Rv (Figure 2).

### CAS1 strains with RD149 deletions show reduced intracellular growth

**Figure 3:**
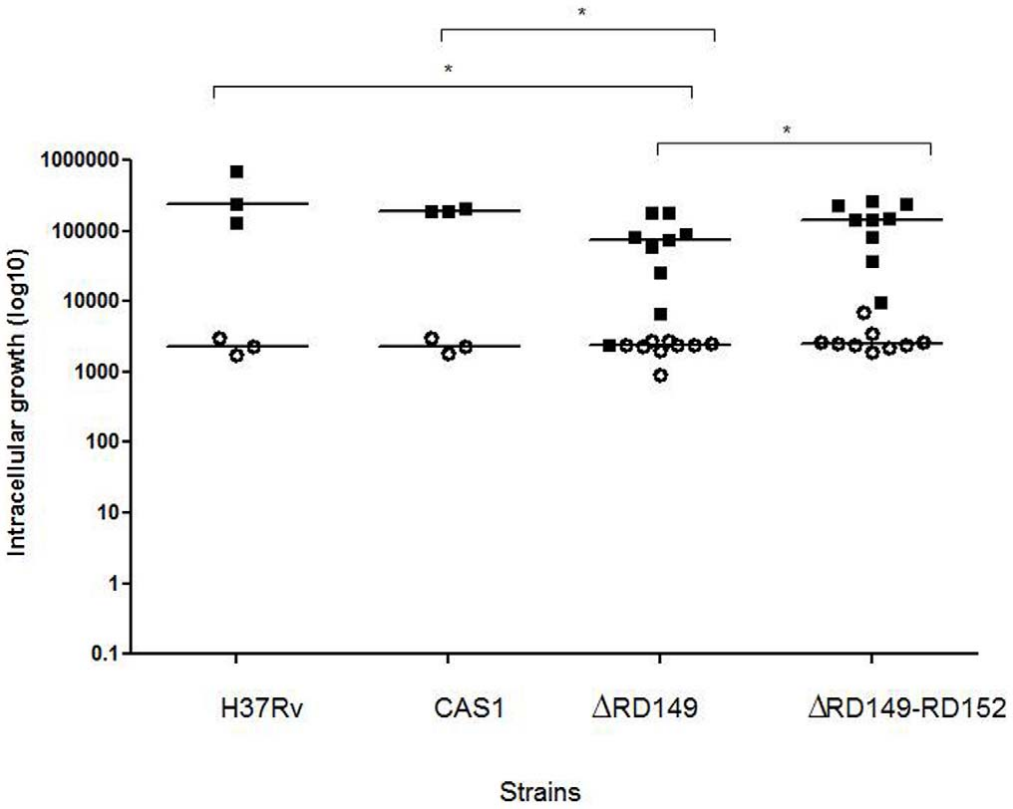
CAS1 strains with RD149 deletions show reduced intracellular growth within THP-1 monocytes. THP-1 (2×10^5^ cells) were infected with *M. tuberculosis* strains (2×10^5^ CFU/ml) and mycobacteria were quantitated at 0 and 3 days. Data is presented as the median of each group as shown by a horizontal bar. ‘*’ denotes significantly reduced growth (p<0.05) as compared with *M. tuberculosis* H37Rv; CAS1 (without deletions) and CAS1 with concurrent RD149-RD152 deletions using Mann-Whitney U test. *Δ* denotes deletions.

Within THP-1 monocytes, CAS1 strains with RD149 deletion strains showed reduced intracellular growth at day 3 post-infection (p = 0.013) in THP-1 cells as compared with CAS1 strains without deletions, and also as compared to H37Rv (p = 0.007), and CAS1 strains with concurrent RD149-RD152 deletion (p = 0.029), Figure 3.

### Increased TNFα and IL10 induction in CAS1 deletion strains

**Figure 4:**
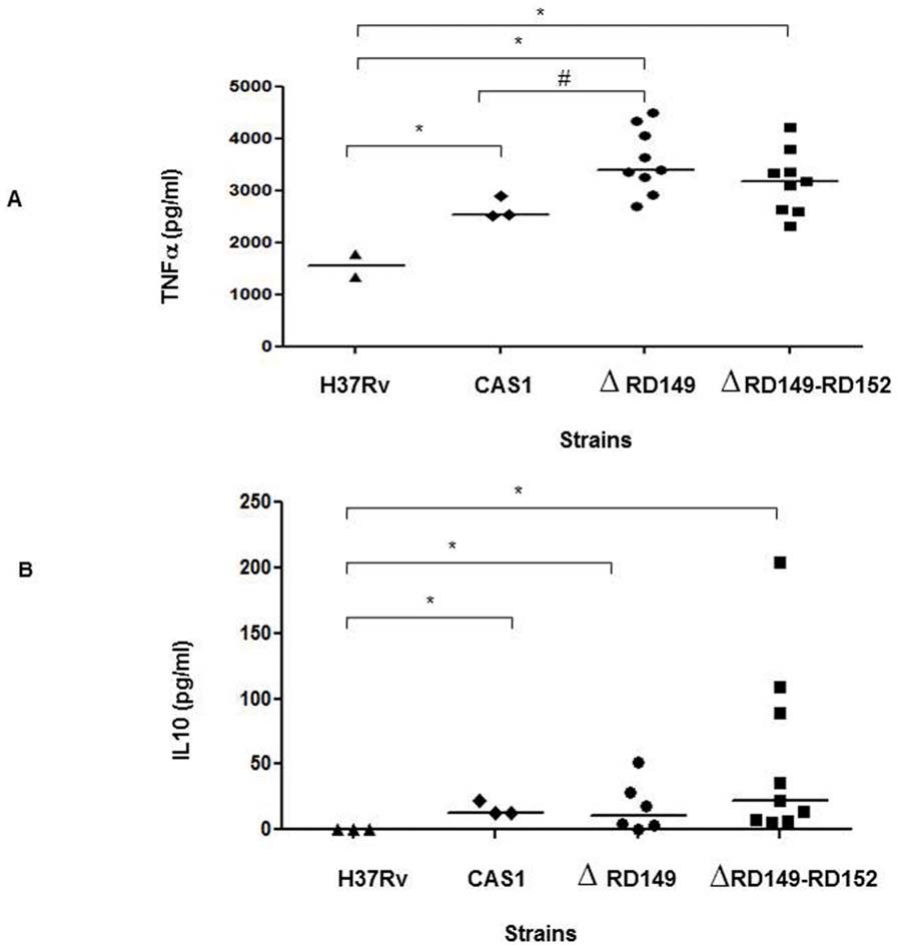
The CAS1 strains with RD149 and concurrent RD149-RD152 deletions induced increased TNFα secretion in THP-1 monocytes. THP-1 (2×10^5^ cells) were infected with *M. tuberculosis* strains (2×10^5^ CFU/ml) and cytokine levels in infected cell supernatants tested for TNFα (18 h) and IL10 (72 h) post infection. ‘*’ (p<0.05) denotes significantly increased cytokine secretion as compared with *M. tuberculosis* H37Rv. ‘#’ (p<0.05) denotes significantly increased cytokine secretion as compared with CAS1 (without deletions) using Kruskal-Wallis test. A horizontal bar indicates median values of each group. Graphs depict cytokines induced by H37Rv, CAS1 (without deletions), CAS1 with RD149 and concurrent RD149-RD152 deletions. *Δ* denotes deletions. A, TNFαand B, IL10.

To investigate association between the presence of deletions and the capacity of *M. tuberculosis* strains to elicit host cytokine activation. TNFα, IL6, IL10 and CCL2 secretion by THP-1 cells infected with CAS1 strains was measured. Significantly higher levels of TNFα induction was noted by CAS1 strains; CAS1 without deletions (p = 0.006), strains with RD149 (p = 0.005) and with concurrent RD149 and RD152 (p<0.001) as compared to *M. tuberculosis* H37Rv. Within CAS1 strains though, isolates with RD149 deletions induced significantly higher levels of TNFα as compared to strains without deletions, (p = 0.013), Figure 4A.

In comparison to *M. tuberculosis* H37Rv, CAS1 strains also induced higher levels of IL10; CAS1 without deletions (p = 0.005), strains with RD149 (p = 0.002) and strains with concurrent RD149-RD152 deletions (p = 0.001), Figure 4B.

Induction of IL6 and CCL2 by the study strains was investigated. However, no differences were detected between IL6 induction by CAS1 strains and H37Rv, or between strains with and without deletions; IL6 levels induced by the study strains were as follows: CAS1 (without deletion); 554 pg/ml, CAS1 strains with RD149 deletions; 526 pg/ml, CAS1 strains with concurrent RD149 and RD152 deletions; 542 pg/ml and *M. tuberculosis* H37Rv; 680 pg/ml. Similarly, CCL2 levels were comparable in all the strains tested: CAS1 (without deletion); 1474 pg/ml, CAS1 with RD149 deletions; 1958 pg/ml, CAS1 with RD149 and RD152 deletions; 2183.5 pg/ml and *M. tuberculosis* H37Rv; 1270 pg/ml.

### Differential intracellular growth of strains from pulmonary and extrapulmonary sources

To investigate whether the source; pulmonary or extrapulmonary had any bearing on strain virulence, growth and cytokine activation in response to strains was compared. No difference was observed in growth rates or activation of host cytokines between pulmonary or extrapulmonary strains of CAS1 or CAS1 with concurrent RD149 and RD152 deletions. In contrast, extrapulmonary strains with RD149 deletions showed increased intracellular growth (p = 0.015) and induced lower levels of TNFα (p = 0.01), CCL2 (p = 0.003) and IL6 (p = 0.008) secretion in THP-1 monocytes as compared with strains from pulmonary sources, Table 2.

**Table 2:** Comparison of growth characteristics and cytokine activation of CAS1 strains from pulmonary and extrapulmonary sources with RD149 and concurrent RD149-RD152 deletions.

	Strains with RD149 deletions			Strains with concurrent RD149-RD152 deletions		
Groups	Pul(n = 4)	Epul(n = 5)	Pvalue	Pul(n = 6)	Epul(n = 3)	Pvalue
Growth in broth##	1.56	1.1	0.373	1.82	1.26	0.201
Growth in THP-1 cells[Table-fn nt107] 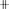	1.31	1.58	0.015*	1.6	1.597	0.19
TNFα (pg/ml)	4153	3164	0.01*	3092	3008	0.767
CCL2 (pg/ml)	2489.5	1306	0.003*	2250	1392	0.153
IL6 (pg/ml)	620.5	314	0.008*	663.5	450	0.051
IL10 (pg/ml)	10.82	14.33	0.643	18.1	36	1.00

CAS1: Central Asian Strain 1 (ST26); Pul (pulmonary source); EPul (extrapulmonary source).

*denotes a significant difference between strains with deletions when compared between median values of Pul and EPul, p<0.05 using Mann-Whitney U test.

## denotes a growth ratio of strain calculated as growth at day 2/ initial growth at day 0.

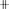
denotes intracellular growth calculated as CFU/ml at day 3/CFU/ml at day 0.

## Discussion

This study showing that presence of RD149 deletions in *M. tuberculosis* CAS1 strains is associated with reduced growth (in broth and *in vitro* macrophages) and with increase in proinflammatory TNFα suggests a role of RD149 in causing phenotypic diversity within these strains.

Earlier studies on growth characteristics of *M. tuberculosis* strains have hypothesized that faster growth of clinical strains such as Beijing in comparison to the laboratory reference H37Rv may contribute to their success in the establishment of disease [8]. Faster growth in broth seen amongst CAS1 strains without deletions as compared with H37Rv in this study may thus be contributing to the success of these strains. However, the presence of RD149 either singly or concurrent with RD152 appears to influence this advantage.

We found that the optimal time period to study intra-strain growth differences was at 3 days post-stimulation. At this time point, CAS1 strains with RD149 deletions showed reduced intracellular growth in THP-1 macrophages as compared with H37Rv, with CAS1 strains (without deletions) and with CAS1 with concurrent RD149-RD152 deletions. This difference was not present at the later 5 day time point. However, the intracellular growth difference observed at the earlier time point is important as it likely to represent early events in mycobacterium-host cell interactions such as, uptake into the phagosome followed by sorting within endosomal compartments of the host cell [33]. Early events post uptake and entry into the macrophage are key to determining phagosome biogenesis and outcome of the infection. Therefore, variations between strains which affect intracellular growth even in an interim period through retardation of the mycobacterial phagosome may well impact host immune response and therefore the outcome of infection.

All CAS1 strains investigated induced higher TNFα as well as IL10 levels in THP-1 cells than the laboratory strain H37Rv. Our data is consistent with reports of increased TNFα and IL10 induction in THP-1 monocytes by clinical strains including the Beijing genotype [8]. TNFα has been shown to play a crucial role in macrophage activation and granuloma formation and thus in controlling mycobacterial replication [34]. TNFα has also been associated with cellular damage and dissolution of the granuloma due to cellular necrosis [34]. While *M. tuberculosis* infection of alveolar macrophages results in TNFα dependent apoptosis [35] and pathogen killing, it may alternatively also lead to the dissemination of the bacillus due to TNFα driven necrosis [36]. It is therefore possible to hypothesize that increased induction of the down modulatory IL10 together with proinflammatory TNFα by CAS1 strains may thus contribute to skewing the host immune response towards a downmodulatory phenotype which would facilitate intra-macrophage growth of the strains.

CAS1 strains with RD149 deletions induced higher TNFα levels as compared to CAS1 strains without deletions. Clinical isolate CDC1551 strain have been shown to induce higher TNFα responses in monocytes but to persist for shorter periods than the more virulent Beijing strain [37]. Similarly, avirulent *Mycobacterium sp*. is reported to induce higher TNFα secretion than the virulent strains [34]. Therefore, the reduced intracellular growth and increased TNFα secretion of CAS1 strains with RD149 deletions may be suggestive of a decrease in virulence amongst the deletion strains.

Beijing strains have been shown to differ in their ability to induce cytokines in both *in vitro* and in animal models [22], [38]. Differences between Beijing strains have been attributed to variation in the production of lipids including Phenolic glycolipids (PGL); strains with mutations in the *pks* 15/1 gene induce higher levels of cytokine-dependent Th1-type protective immune responses such as TNFα [7], [39]. Recent studies have identified variations in the transcriptome profiles of *M. tuberculosis* strains from different lineages suggesting, that genetic variations may impact phenotypic patterns of clinical strains [40]. It is likely therefore that the differences observed in responses to CAS1 strains could be due to inter-strain variations perhaps even due to the presence of RD deletions.

RD149 and RD152 are associated with mobile genetic elements e.g., prophage and hot spots for IS6110 insertion, respectively [41]. The *Rv 1759c* gene (*wag22*) within RD152 region is a member of the PE family, polymorphic CG-repetitive sequences (PGRS) subfamily of glycine rich proteins which are putative virulence factors for *M. tuberculosis* [41]. The combined effect of RD149 and RD152 deletions may differ from that of RD149 deletion strains due to difference in strain properties which result from the variable antigenic profile of strains as a result of their differing presentations. This may subsequently affect uptake and intracellular growth of strains hence, the differential outcome of infections with RD149 strains from that of strains with RD149-RD152.

Within CAS1 strains with RD149 deletions, those from extrapulmonary sites grew faster and induced lower levels of TNFα, CCL2 and IL10 as compared with pulmonary strains. This is consistent with trends from a previous study showing that extrapulmonary *M. tuberculosis* strains grow faster and induce less TNFα secretion in human monocyte derived macrophages [42].

To date, there is little data on the role of RD149 and RD152 deletions in either the virulence or the epidemiology of TB. The current study comparing clinical strains with and without RD deletions suggests that observed differences in growth and host cytokine modulation are associated with deletions in RD149. We hypothesize that the variations observed in the virulence patterns of *M. tuberculosis* clinical isolates may be affected by the acquisition of RD149 deletions and may further be dependent on the location of the deletions on the bacterial chromosome. This is important since the data suggests that these deletions may have an impact on pathogenesis and therefore on clinical outcome of TB infection.

## Supporting Information

Figure S1 **Measurement of** ***M. tuberculosis*** **growth in broth using the BACTEC system.** *M. tuberculosis* H37Rv was adjusted to approx cell density of 1.5×10^6^ bacterial cells/ml for inoculation and subsequently grown for 37 days in the radiometric BACTEC 460 TB system. To determine Colony Forming Units (CFUs), bacterial suspension from 12B medium was plated on 7H10 agar medium and incubated for 3 weeks for enumeration. Growth in broth was measured for H37Rv, CAS1 strains (without deletions), CAS1 strains: S1, S2, S5, S6, EP1, EP2, EP4, EP7 and EP10 with RD149 deletions and CAS1 strains: S3, S4, S7, S8, S9, S10, EP6, EP8 and EP9 with concurrent RD149-RD152 deletions at days: 0, 2, 7, 12, 17, 22, 27, 32 and 37. Data shown is the mean of two independent experiments with ‘y’ error bars indicating standard deviation is illustrated in Figure S1. All experiments were repeated twice (with duplicates in each experiment). The data presented is the mean of 2 experiments.
(TIF)

Figure S2 **Intracellular growth of** ***M. tuberculosis*** **growth in THP-1 monocytes.** The THP-1 cells (2×10^5^ cells/well) were infected with *M. tuberculosis* H37Rv at (2×10^5^ CFU/ml) and cultured for upto 7 days. Graph depicts mycobacterial numbers harvested from cells upon lysis of infected monolayers at 0, 1, 3 and 5 days post infection. The intracellular growth (log_10_) was measured for H37Rv, CAS1 strains (without deletions), CAS1 strains: S1, S2, S5, S6, EP1, EP2, EP4, EP7 and EP10 with RD149 deletions and CAS1 strains: S3, S4, S7, S8, S9, S10, EP6, EP8 and EP9 with concurrent RD149-RD152 deletions at days 0, 1, 3 and 5. Data shown is the mean of three independent experiments with ‘y’ error bars indicating standard deviation is illustrated in Figure S2. All experiments were performed in triplicate and the data presented is the mean of 3 experiments.
(TIF)
