# Supplementary figures and images for: Presence of RD149 Deletions in M. tuberculosis Central Asian Strain1 Isolates Affect Growth and TNFα Induction in THP-1 Monocytes
Source: PLoS One. 2011 Aug 31;6(8):e24178. doi: 10.1371/journal.pone.0024178 (PMC3163664; doi:10.1371/journal.pone.0024178)

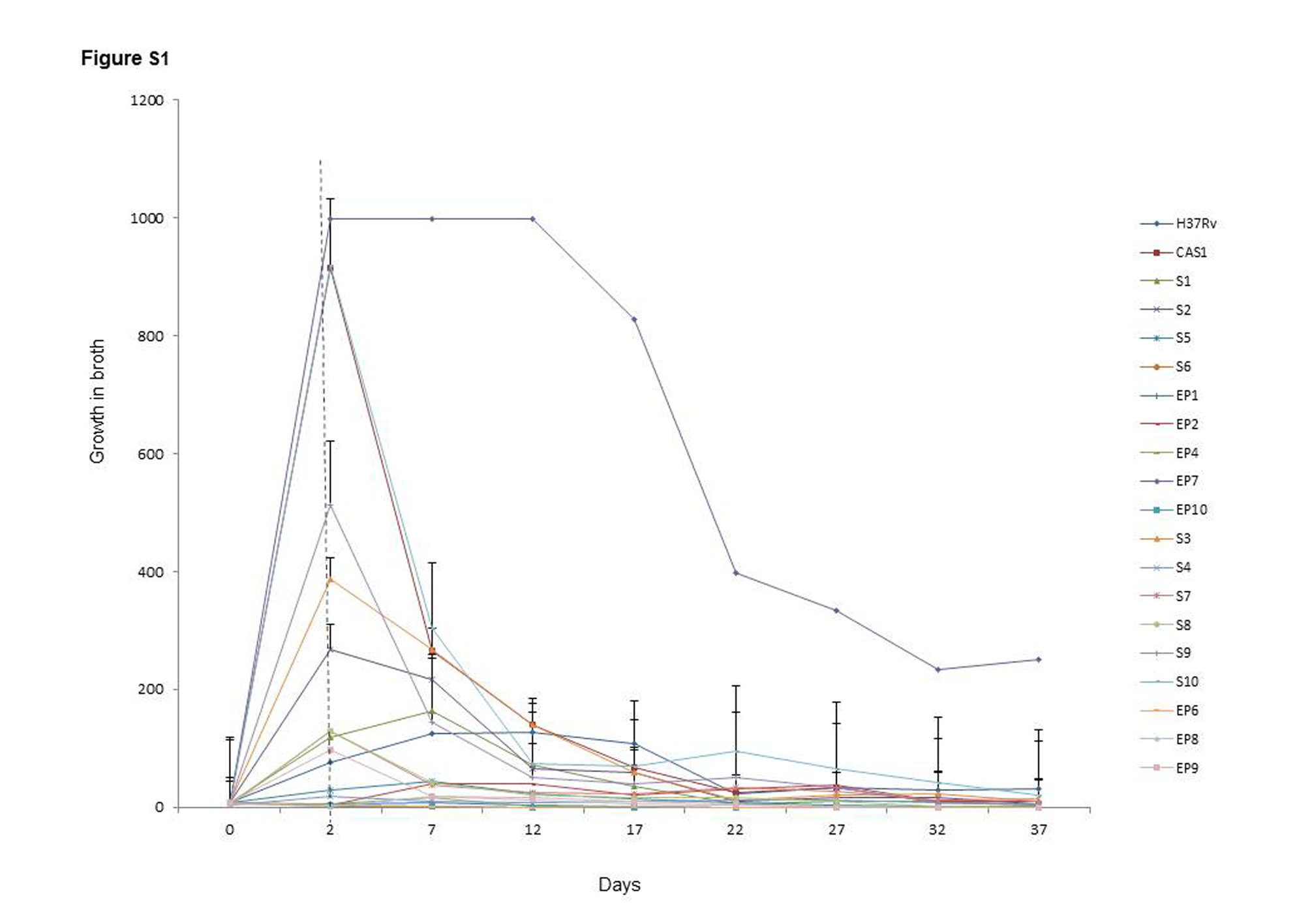

Supplement: Figure S1 — Measurement of M. tuberculosis growth in broth using the BACTEC system. M. tuberculosis H37Rv was adjusted to approx cell density of 1.5×10^6^ bacterial cells/ml for inoculation and subsequently grown for 37 days in the radiometric BACTEC 460 TB system. To determine Colony Forming Units (CFUs), bacterial suspension from 12B medium was plated on 7H10 agar medium and incubated for 3 weeks for enumeration. Growth in broth was measured for H37Rv, CAS1 strains (without deletions), CAS1 strains: S1, S2, S5, S6, EP1, EP2, EP4, EP7 and EP10 with RD149 deletions and CAS1 strains: S3, S4, S7, S8, S9, S10, EP6, EP8 and EP9 with concurrent RD149-RD152 deletions at days: 0, 2, 7, 12, 17, 22, 27, 32 and 37. Data shown is the mean of two independent experiments with ‘y’ error bars indicating standard deviation is illustrated in Figure S1. All experiments were repeated twice (with duplicates in each experiment). The data presented is the mean of 2 experiments. (TIF) [file pone.0024178.s001.tif]

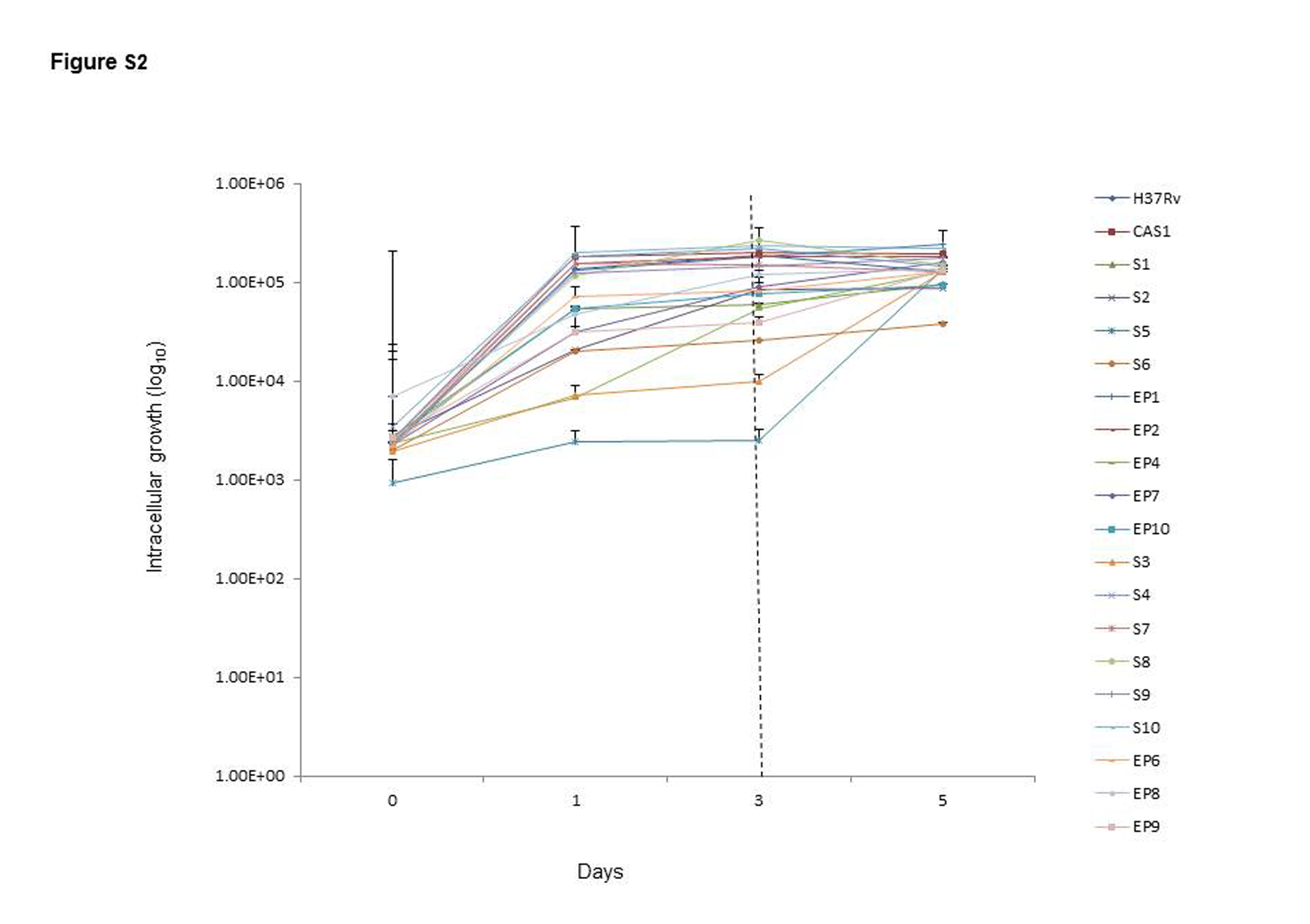

Supplement: Figure S2 — Intracellular growth of M. tuberculosis growth in THP-1 monocytes. The THP-1 cells (2×10^5^ cells/well) were infected with M. tuberculosis H37Rv at (2×10^5^ CFU/ml) and cultured for upto 7 days. Graph depicts mycobacterial numbers harvested from cells upon lysis of infected monolayers at 0, 1, 3 and 5 days post infection. The intracellular growth (log10) was measured for H37Rv, CAS1 strains (without deletions), CAS1 strains: S1, S2, S5, S6, EP1, EP2, EP4, EP7 and EP10 with RD149 deletions and CAS1 strains: S3, S4, S7, S8, S9, S10, EP6, EP8 and EP9 with concurrent RD149-RD152 deletions at days 0, 1, 3 and 5. Data shown is the mean of three independent experiments with ‘y’ error bars indicating standard deviation is illustrated in Figure S2. All experiments were performed in triplicate and the data presented is the mean of 3 experiments. (TIF) [file pone.0024178.s002.tif]
